# The role of Chinese herbal medicine in the regulation of oxidative stress in treating hypertension: from therapeutics to mechanisms

**DOI:** 10.1186/s13020-024-01022-9

**Published:** 2024-10-29

**Authors:** Zixuan Jin, Yu Lan, Junying Li, Pengqian Wang, Xingjiang Xiong

**Affiliations:** 1 Department of Cardiology, Guang’anmen Hospital, China Academy of Chinese Medical Sciences, No.5 Beixian Ge, Xicheng District, Beijing, 100053 China; 2 Institute of Chinese Materia Medica, China Academy of Chinese Medical Sciences, Beijing, 100700 China

**Keywords:** Traditional Chinese medicine, Chinese herbal medicine, Oxidative stress, Hypertension

## Abstract

**Background:**

Although the pathogenesis of essential hypertension is not clear, a large number of studies have shown that oxidative stress plays an important role in the occurrence and development of hypertension and target organ damage.

**Purpose:**

This paper systematically summarizes the relationship between oxidative stress and hypertension, and explores the potential mechanisms of Chinese herbal medicine (CHM) in the regulation of oxidative stress in hypertension, aiming to establish a scientific basis for the treatment of hypertension with CHM.

**Methods:**

To review the efficacy and mechanism by which CHM treat hypertension through targeting oxidative stress, data were searched from PubMed, EMBASE, the Cochrane Central Register of Controlled Trials, the Chinese National Knowledge Infrastructure, the VIP Information Database, the Chinese Biomedical Literature Database, and the Wanfang Database from their inception up to January 2024. NPs were classified and summarized by their mechanisms of action.

**Results:**

In hypertension, the oxidative stress pathway of the body is abnormally activated, and the antioxidant system is inhibited, leading to the imbalance between the oxidative and antioxidative capacity. Meanwhile, excessive production of reactive oxygen species can lead to endothelial damage and vascular dysfunction, resulting in inflammation and immune response, thereby promoting the development of hypertension and damaging the heart, brain, kidneys, blood vessels, and other target organs. Numerous studies suggested that inhibiting oxidative stress may be the potential therapeutic target for hypertension. In recent years, the clinical advantages of traditional Chinese medicine (TCM) in the treatment of hypertension have gradually attracted attention. TCM, including active ingredients of CHM, single Chinese herb, TCM classic formula and traditional Chinese patent medicine, can not only reduce blood pressure, improve clinical symptoms, but also improve oxidative stress, thus extensively affect vascular endothelium, renin–angiotensin–aldosterone system, sympathetic nervous system, target organ damage, as well as insulin resistance, hyperlipidemia, hyperhomocysteinemia and other pathological mechanisms and hypertension related risk factors.

**Conclusions:**

CHM display a beneficial multi-target, multi-component, overall and comprehensive regulation characteristics, and have potential value for clinical application in the treatment of hypertension by regulating the level of oxidative stress.

## Introduction

In recent years, the incidence rate of hypertension has increased year by year, often accompanied by damage to heart, brain, kidney, eyes and other important target organs [1], which has become a major global public health problem. Although the pathogenesis of hypertension is not yet clear, extensive research has confirmed that oxidative stress plays an important role in the occurrence and development of hypertension and its complications.

Oxidative stress is the oxidative damage to tissues and organs caused by the imbalance of redox reaction in the body. Numerous studies have shown that oxidative stress is an important mechanism for the occurrence and development of hypertension and its target organ damage (TOD) complications [2]. Under physiological conditions, the human body has a complete antioxidant defense system that can eliminate oxygen free radicals and reactive oxygen species (ROS) produced by tissue and organ metabolism, playing a role in inhibiting diseases and delaying aging [3]. The human antioxidant system is composed of various antioxidant enzymes and non enzymatic antioxidant substances. Antioxidant enzymes include superoxide dismutase (SOD), catalase (CAT), glutathione peroxidase (GSH-Px), etc. Non-enzymatic antioxidants include vitamins C, vitamins E, tea polyphenols, carotenoids, resveratrol, etc. The enzyme and non enzyme antioxidant systems in the body are interdependent and complementary, working together to combat oxidative substances produced in the body and exert antioxidant effects. Under physiological conditions, the body is in a state of balance between oxidation and antioxidant. However, in pathological conditions, the body's oxidative and antioxidant abilities are imbalanced. On the one hand, the body's oxidative capacity is enhanced, and a large amount of ROS is produced. On the other hand, the antioxidant system is weakened and the synthesis of antioxidant substances is insufficient. Excessive oxidative substances cannot be promptly cleared and stored in the body by the antioxidant system, leading to oxidative damage to tissues and organs, dysfunction of vascular endothelial cells, abnormal activation of the renin angiotensin aldosterone system (RAAS) and sympathetic nervous system (SNS), proliferation of vascular smooth muscle cells, exacerbation of vascular inflammation, sympathetic nervous system excitation, and vascular remodeling, ultimately resulting in the occurrence and development of various diseases including hypertension.

**Fig. 1:**
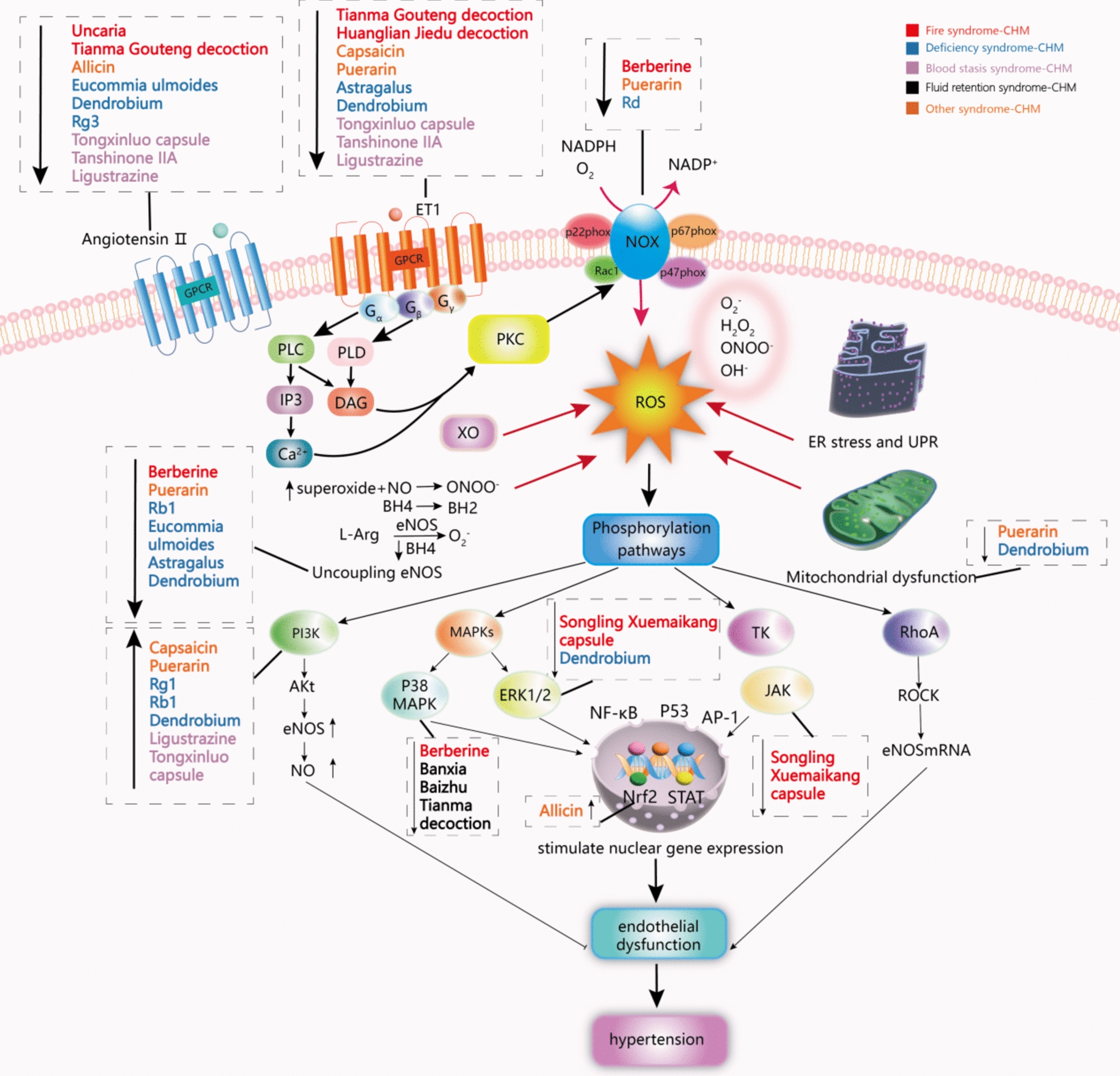
The role of Chinese herbal medicine (CHM) in the regulation of oxidative stress in treating hypertension. “↑” means upregulated; “↓” means downregulated

In traditional Chinese medicine (TCM), extensive experience and theory in the diagnosis and treatment of hypertension has been accumulated. Hypertension can be divided into many subtypes based on the classification criteria of syndromes rather than symptoms as population characteristics, including fire syndrome, fluid retention syndrome, deficiency syndrome, and blood stasis syndrome [4]. Taking fire syndrome as an example, it includes clinical manifestations such as dizziness, headache, restlessness, irritability, insomnia, dreams, red tongue, and rapid pulse, which is significantly different from the clinical manifestations of fluid retention syndrome, deficiency syndrome, and blood stasis syndrome. In terms of pathophysiology, it is not only closely related to inflammation, increased sympathetic activity, oxidative stress damage, but also closely related to the ROS/Akt oxidative stress pathway and the nitric oxide cyclic guanosine monophosphate signaling pathway [5]. In the clinical treatment of fire syndrome, the commonly used herbal medicine included Berberine, Uncaria, *Tianma Gouteng* deccision, *Huanglian Jiedu* deccision, Songling Xuemaikang capsule, etc. The classification and treatment approach based on syndrome has brought new ideas for the development of new drugs for hypertension. Studies have confirmed that TCM can stabilize blood pressure and reverse risk factors in the treatment of hypertension [4, 5]. In addition, modern pharmacological and clinical studies have shown that TCM plays an important regulatory role in the inhibition of oxidative stress in the treatment of hypertension. Chinese herbal medicine (CHM), including single Chinese herbal medicine, TCM classic formula and traditional Chinese patent medicine, can reduce blood pressure and protect target organs through oxidative stress injury (Fig. 1). This article will discuss the relationship between oxidative stress and hypertension, and the mechanism of CHM in regulating oxidative stress in the treatment of hypertension.

## Oxidative stress and hypertension

### Oxidative stress and vascular endothelial injury

Endothelial injury and dysfunction of vascular contraction and relaxation are important pathological foundations for the occurrence and development of hypertension. The endothelium of blood vessels can secrete various vasoactive substances, which play an important role in maintaining blood pressure stability. Oxidative stress is a key mechanism leading to endothelial dysfunction. These vasoactive substances are composed of endogenous relaxation factors and endogenous systolic factors. The former includes nitricoxide (NO), and the latter includes ROS, angiotensin II (Ang II), endothelin1 (ET-1), etc. [6].

Endothelial nitric oxide synthase (eNOS) is mainly distributed in vascular endothelial cells and is a key enzyme in endothelial NO synthesis, leading to the synthesis of endothelial NO. NO has antioxidant, vasodilatory, preventive effects on platelet and inflammatory cell adhesion and aggregation, and inhibition of vascular smooth muscle proliferation. The ROS synthesis system of the vascular wall includes reduced coenzyme II (nicotinamide adenine dinucleotide phosphate, NADPH) oxidase, xanthine oxidase (XOD), mitochondria, and uncoupled eNOS [7, 8]. Among them, NADPH oxidase can decouple eNOS [9], activate XOD and promote the production of ROS in mitochondria [10, 11], which may play a role in constricting blood vessels, exacerbating oxidative stress, and endothelial damage.

Under physiological conditions, there is a dynamic balance between endogenous contraction factors and endogenous relaxation factors, which maintains the coordination of vasomotor function and the balance between oxidation and antioxidation. Elevated blood pressure can lead to an increase in ROS production and a decrease in NO synthesis in the endothelium of blood vessels, resulting in dysfunction of vascular wall contraction and relaxation, and exacerbating oxidative stress. Oxidative stress in blood vessels, in turn, can further exacerbate endothelial cell dysfunction, as well as the infiltration and activation of inflammatory cells, forming a malignant positive feedback mechanism [12]. Experimental studies have shown that in the spontaneously hypertensive rat (SHR) model, the activity of NADPH oxidase and high levels of O2- in the vascular wall can lead to an increase in ROS production and a decrease in endogenous NO production, resulting in cell and tissue damage [13].

### Oxidative stress and abnormal activation of renin–angiotensin–aldosterone system (RAAS)

The RAAS system plays an important role in regulating water and sodium metabolism and reversing myocardial remodeling. The abnormal activation of the RAAS system is one of the important mechanisms underlying the occurrence and development of hypertension. The RAAS system consists of ACE/ANGII/AT1 axis and ACE2/ANG1-7/mas axis.

Renin is the promoter of ACE/AngII/AT1 axis, which can degrade Angiotensinogen in blood to Angiotensin I (Ang I). Ang I is further converted into angiotensin II (AngII) in lungs. Ang II binds to its receptor AT1R, causing vasoconstriction, increased synthesis of pro-inflammatory cytokines, and increased secretion of aldosterone. In the ACE2/Ang1-7/mas axis, angiotensin converting enzyme 2 (ACE2) can degrade the action product Ang II of angiotensin-converting enzyme (ACE), leading to the production of Ang-(1–7). Meanwhile, ACE2 can competitively act on AngI to generate Ang-(1–9), which in turn generates Ang-(1–7). Ang-(1–7) binds to Mas receptors and exerts vasodilation and antioxidant effects. Under physiological conditions, the ACE2/Ang1-7/mas axis and ACE/AngII/AT1 axis mutually constrain and antagonize each other, maintaining dynamic balance and jointly ensuring stable blood pressure. However, the ACE/AngII/AT1 axis in hypertensive patients is overactivated, disrupting the balance between the ACE2/Ang1-7/mas axis and the ACE/AngII/AT1 axis, which further exacerbates the occurrence and development of hypertension and its complications.

The ACE/AngII/AT1 axis can promote oxidative stress and release of inflammatory cytokines in SHR. The decrease in AngII/Ang-(1–7) ratio can reduce ROS levels and enhance antioxidant stress resistance [14]. In vivo and in vitro studies on Ang II induced myocardial hypertrophy in neonatal rats have shown that AngII increases the levels of malondialdehyde (MDA) and SOD, leading to myocardial hypertrophy and remodeling. Meanwhile, Mas receptor mediated Ang-(1–7) has a cardioprotective effect on Ang II induced cardiomyocyte autophagy and cardiac remodeling by inhibiting oxidative stress [15]. In addition, AngII can activate NADPH oxidase, up-regulate ROS levels and the expression of pro-inflammatory cytokines, thereby further activating oxidative stress and the expression of several components in the RAAS system, forming a vicious cycle of positive feedback mechanism [16].

### Oxidative stress and abnormal activation of the SNS

Abnormal activation of SNS is an important factor in the occurrence and development of hypertension. The hyperfunction of SNS and the massive release of catecholamine neurotransmitter norepinephrine in the blood are the main mechanisms of sympathetic nervous system-mediated hypertension [17].

Norepinephrine is the main material basis for the SNS to play its role in the regulation of cardiovascular function. When the SNS is excited, sympathetic vesicles release norepinephrine and bind to the corresponding adrenergic receptors to exert their effects. Adrenergic receptors can be divided into α-receptors and β-receptors, which are specific targets of norepinephrine to mediate the pathophysiological effects. The distribution of α and β receptors is different in different tissues. The binding of catecholamine neurotransmitters to α receptors leads to vasoconstriction, while the binding to β1 receptors increases heart rate and cardiac output. The activation of both receptors can lead to the occurrence and development of hypertension [18]. A meta analysis involving 524 patients from 6 renal denervation studies showed that the SNS activation affects blood pressure variability (BPV), and new interventional techniques for renal denervation can regulate SNS activation, reducing the standard deviation of 24-h systolic hypertension pressure (SBP) and 24-h diastolic blood pressure (DBP) standard, which impacted on the short time blood pressure variability (BPV) of hypertensive patients [19].

Oxidative stress and SNS can interact and promote each other. The chronic cardiac overload model in rats is characterized by SNS activation, leading to increased RAAS activity and aggravated oxidative stress. Renal denervation can reduce RAAS activity and oxidative stress levels in rats [20]. For SHR induced by high fat diet, transcatheter radiofrequency ablation of renal sympathetic nerve can continuously reduce SBP and DBP, lower serum AngII, NADPH oxidase and MDA levels at various postoperative time points, increase eNOS and NO content, thereby reducing SNS activity, inhibiting oxidative stress, and treating hypertension [21]. Therefore, blocking the renal sympathetic nervous system can to some extent inhibit oxidative stress.

## Role of CHM in the regulation of oxidative stress for hypertension

### Active ingredients of CHM

#### Ginsenoside

Ginseng is the root of Panax ginseng. According to TCM theory, ginseng has a mild nature, a sweet and bitter taste, and belongs to the lungs, spleen, heart and kidney meridians. It has the effects of reinforcing the vital energy, reinforcing the arteries, reinforcing the spleen, nourishing the lung, nourishing the blood, calming the mind, and improving the intelligence. Modern pharmacological studies have shown that ginseng has pharmacological effects of antihypertensive, antioxidant [22], anti-allergy, hypoglycemic, anti-inflammatory, anti-cancer [23], anti-aging [24], anti-fatigue [25], anti-apoptosis, inhibition of oxidative stress, and angio-modulation[26], etc., which is often used for clinical treatment of hypertension, diabetes, allergic diseases, chronic fatigue syndrome, cancer and other diseases. Ginseng not only lowers blood pressure, but also has a wide range of cardiovascular protective effects, such as antioxidant, improving vasodilation and vasoconstriction function, improving lipid distribution, and reducing platelet adhesion [27]. A meta-analysis of 528 hypertensive patients from 9 RCTs showed that ginseng can reduce SBP and DBP by 6.52 mmHg and 5.21 mmHg, respectively [28]. A RCT involving 62 patients with prehypertension showed that after 12 weeks of treatment, ginseng reduced SBP and DBP by 6.5 mmHg and 5.0 mmHg, respectively. The pharmacological mechanism is related to the downregulation of lipoprotein-associated phospholipase A2 (Lp-PLA2) and lysophosphatidylcholines (lysoPCs) levels, as well as the upregulation of dihydrobiopterin levels [29].

Ginsenosides (Table 1) are the main active ingredients of ginseng. At present, there are about 150 known ginsenosides, including Rb1, Rb2, Rc, Rd, Re, Rg1, Rg3, etc. [30]. Multiple studies have shown that ginsenosides have a certain antihypertensive effect. A RCT of 80 hypertensive patients with type 2 diabetes showed that after 12 weeks of combined administration of American Ginseng (AG) and Korean Red Ginseng (KRG) rich in Rg3, the central SBP and the final SBP can be reduced by 4.69 mmHg and 6.60 mmHg, respectively, suggesting that Rg3 has a certain antihypertensive effect [31]. In the SHR experimental model, after 8 weeks of treatment, taking 1000 mg/kg of hypotensive components-enriched fraction of red ginseng can decrease SBP and DBP by 17% and 11%, respectively [32]. In addition, Rg3 can decrease the level of AngII by up-regulating the level of ACE2 in renal tissue, which could inhibit the progression of hypertensive nephropathy [33].

**Table 1:** In vitro and *vivo* protective effects of active ingredients of CHM in the regulation of oxidative stress for hypertension

Source	Active component	Structure	Animal/cells (dosage)	Targets	Refs.
*Renshen* (Ginseng)	Ginsenoside		SHR (oral administration, 1000 mg/kg/d, 8 weeks), 2K2C (oral administration, 80 ng/ml, 2 h), NRK-52E cells (pre-incubate with Rg1, 80 ng/ml, 2 h), and HUVEC (10 or 20 µM, 30 min)	AngII↓, ACE2↑, SIRT1/AMPK/eNOS/NO↑, Nox1↓, Nox2↓, PI3K/Akt/eNOS↓	[32, 34–36]
*Dasuan* (Garlic)	Allicin		Cardiac hypertrophy rat (oral administration, 180 mg/kg/d, 8 weeks), CKD rat (oral administration, 40 mg/kg/d, 6 weeks), metabolic syndrome rat (oral administration, 50 mg/kg/d, 8 days), and SHR (Intraperitoneal injection, 7 mg/kg/d or 14 mg/kg/d, 4 weeks)	AT1R↓, KEAP1↓, TC↓, Hcy↓, SCR↓, BUN↓, CCR↑, CAT↑, Nrf2↑, NQO1↑, γ-GCS↑, SOD↑, eNOS↑	[37],García et al., 2017; [2, 38]
*Lajiao* (Capsicum)	Capsaicin		2K1C (oral administration, 0.006% capsaicin diet, 6 weeks ), guinea pigs (oral administration, 10 mg/kg/d, 14 weeks), SD rat (subcutaneous injection, 100 mg/kg/d, 5 days ), SHR (oral administration, 20 mg/kg/d, 6 weeks), and erythrocytes (oral administration, 20 mg/kg/d, 21 days)	Akt↑, TRPV1↑, TC↓, TG↓, LDL-C↓, HDL-C↑, ET-1↓, AGV↓, SOD↑, NO↑, MDA↓, GSH↑, ROS↓, eNOS↑	[39, 39–42]
*Huanglian* (Rhizoma coptidis)	Berberine		diabetic rat (intragastric administration, 50, 100 and 200 mg/kg/d, 8 weeks), SHR (intragastric administration, 50 mg/kg/d, 4 weeks), and 2K1C (infusion, 2 μg/h, 28 days)	BKCA↑, EMPs↑, EPCs↑, PI3K/Akt↓, p38MAPKs↓, ACE↓, TRPV4↓, NADPH oxidase↓, NE↓, COX-2↓, AMPK↑, ROS/ERK1/2/iNOS↓, NO/cGMP↑, NOX2↓, NOX4↓	[43–45]
*Gegen* (Pueraria)	Puerarin		hypersaline-induced hypertensive mice (intragastric administration, 30 mg/kg/d, 4 weeks), AngII-induced hypertensive rat (intragastric administration, 100 mg/kg/d, 10 days), SHR (intraperitoneal injection, 40 or 80 mg/kg/d, 3 months), and HUVEC (1,5,10,25 and 50 g/mL, 48 h)	cGMP↑, AT1↓, TRPV4↑, NADPH↓, TGF-β1↓, MDA↓, PI3K/Akt↓, NO↑, eNOS↑	[46–49]
*Danshen* (Salvia miltiorrhiza)	Tanshinone IIA		2K2C (oral administration, 70, 35 mg/kg/d, 6 weeks) and hypertensive rats (oral administration, 2.15 g/kg/d, 16 weeks)	MMPs/TIMPs↓, Cys-C/Wnt↓, MEK/ERK↓, Gp91↓, IL-1β↓, IL-10↑	Feng et al., 2017; [50]
*Chuanxiong* (Ligusticum chuanxiong)	Ligustrazine		SHR (oral administration, 15, 30 and 60 mg/kg, 4 weeks), 2K2C(oral administration, 25 and 100 mg/kg, 12 weeks), and HUVEC (50, 100 or 150 microg/ml, 24 h)	ET-1↓, AngII↓, NO↑, GSH↑, ROS↓	[51–53]

Previous studies have confirmed that the hypotensive mechanism of ginsenoside is related to the inhibition of oxidative stress. Ginsenoside Rb1 can up-regulate eNOS and NO levels by activating the SIRT1/AMPK pathway, and prevent senescence of human umbilical vein endothelial cells (HUVEC) induced by H_2_O_2_^−^ [34]. In 2 kidneys and 2 clamps rats (2K2C) prone to renal vascular stroke and hypertensive rats induced by AngII, ginsenoside Rd can reduce the expression and activity of Nox1 and Nox2 proteins in NADPH oxidase subunits, inhibit AngII induced NO production, and reduce ROS production, which in turn improve vascular endothelial damage in hypertension [35]. Using renal tubule cells of NRK-52E rats as experimental model, ginsenoside Rg1 can reduce the autophagy and ROS production induced by aldosterone (ALD). The mechanism is related to the regulation of AMPK/mTOR pathway in NRK-52E cells [36]. Ginsenoside Rb1 and Rg1 can also up-regulate the level of NO in the vascular endothelium of SHR by regulating PI3K/Akt/eNOS pathway, thereby playing a role in inhibiting oxidative stress and lowering blood pressure in SHR [54].

#### Allicin

Garlic is an underground bulb of Allium in Liliaceae, which is used for both medicine and food. According to TCM theory, garlic is warm in property, pungent in taste, and belongs to spleen, stomach and lung meridians. Garlic has the effects of detoxification, anti-inflammatory, insecticidal, antidiarrheal, nourishing qi, removing blood stasis, warming the stomach and strengthening the spleen. Modern pharmacological studies have shown that garlic has the pharmacological effects of lowering blood pressure, lowering blood lipids, lowering blood sugar [55], improving renal function, regulating gut microbiota [56], etc. Numerous studies have confirmed that garlic has a certain antihypertensive effect. A meta-analysis of 20 RCTs with 970 hypertensive patients showed that garlic can reduce SBP and DBP by an average of 5.1 mmHg and 2.5 mmHg, respectively [57]. Another meta-analysis of 7 RCTs with 391 hypertensive patients identified that garlic can reduce SBP by 6.71 mmHg, and DBP by 4.79 mmHg, without serious adverse events [58]. Another systematic review of 18 RCTs with 1069 patients revealed that garlic can decrease the level of low-density lipoprotein cholesterol (LDL-C) [59].

Allicin (Table 1) is a trithioallyl ether compound, naturally occurring in the bulb of the Liliaceae plant garlic. It is the main active ingredient of garlic. Allicin has pharmacological effects such as lowering blood pressure [60], antibacterial properties [61], lowering blood lipids [62], lowering blood sugar [63], protecting target organ of the heart [64], lowering body weight, and protecting vascular endothelium [65]. Previous studies have confirmed that allicin has a certain antihypertensive effect. After 6 weeks of treatment with allicin by 40 mg/kg/d/p.o., SBP was decreased by 40 mmHg in chronic kidney disease (CKD) rats. The mechanism may be related to the down-regulation of AT1R and KEAP1 expression, as well as the inhibition of oxidative stress [66]. S-allyl mercaptocaptopril (CPSSA) is a novel and stable synthetic compounds produced through the chemical reaction between allicin and angiotensin converting enzyme inhibitors captopril. Treatment with CPSSA 50 mg/kg/d for 8 days could reduce SBP by 25 mmHg in rats with metabolic syndrome induced by fructose, and its antihypertensive effect is similar to that of captopril. Meanwhile, total cholesterol (TC) and homocysteine (Hcy) can also be reduced [37], suggesting that allicin has a certain antihypertensive effect.

Several studies have shown that the antihypertensive mechanism of allicin is related to the inhibition of oxidative stress. In CKD rats, allicin not only decreases SBP by 10 mmHg, but also lower the levels of serum creatinine (SCR) and blood urea nitrogen (BUN) by 0.308 mg/dL and 20.98 mg/dL, respectively, and increase creatinine clearance (CCR) by 0.299 ml/min. The mechanism may be related to the down-regulation of AngiotensinII Type 1 Receptor (AT1R) level, and the up-regulation of levels of CAT, SOD and eNOS [67]. In AngII-induced cardiac hypertrophy rats, intervention with allicin by 180 mg/kg/d for 8 weeks could not only reverse the decrease of GPX activity induced by AngII, but also increase reactive oxygen content. In addition, the mRNA expression and protein levels of Nrf2, NQO1 and γ-GCS were significantly increased, while left ventricular weight and left ventricular end-diastolic diameter decreased. The antihypertensive mechanism of allicin may be related to the improvement of cardiac hypertrophy by enhancing the Nrf2 antioxidant signaling pathway, as well as the prevention and reversal of cardiac TOD induced by hypertension [38].

Allicin is composed of various sulfur-containing compounds, and hydrogen sulfide (H2S) has been shown to have specific vasomotor effects. In vivo and in vitro studies have shown that allicin can exert endothelium-dependent vasodilation by inducing H2S production, which is related to the mediation of nitric oxy-soluble guanidate-cyclase-cyclic guanosine phosphate (NO-SGC-cGMP), prostacyclin-adenylate cyclase-cyclic adenosine phosphate (PGI2-ac-cAMP) and endothelium-derived hyperpolarizing factor (EDHF), leading to a decrease in blood pressure in SHR [2]. Using SHR as the experimental model, allicin can increase the levels of SOD, GSH and CAT, and decrease the levels of MDA and superoxide anion in SHR model [68]. A number of experimental studies have shown that allicin can inhibit oxidative stress, improve myocardial fibrosis and ventricular remodeling caused by hypertension. The mechanism may be related to the inhibition of the abnormal increase of AngII induced by hypertension and the abnormal activation of TGF-β1 signaling pathway, reduction of MDA level, increase of SOD level, inhibition of proliferation of mouse embryonic fibroblasts (NIH3T3), reduction of collagen secretion and the expression of type 1 collagen [69].

#### Capsaicin

Capsicum is one year or limited perennial herb for Magnolia, Solanaceae, or Capsicum. As a popular condiment, capsicum has a history of more than 100 years in China. According to TCM theory, capsicum is hot in property, pungent in taste, and have the effects of warming the middle, dispersing cold, dispelling qi, and digesting food. It could be used to treat stomach cold stagnation, abdominal distension pain, vomiting, diarrhea, rheumatism pain and chilblain. According to modern medicine, capsicum has pharmacological effects such as antioxidant [70], anti-inflammatory [71], hypoglycemic [72], anti-cancer [73], etc. Numerous studies have confirmed that capsicum has a certain effect of lowering blood pressure. A systematic review of 363 hypertensive patients, including 7 RCTs showed that capsicum could reduce DBP by 1.90 mmHg, suggesting that capsicum can improve blood pressure levels in hypertensive patients [74]. Using adult male Wistar rats as experimental model, capsicum can inhibit the increasing of alanine aminotransferase (ALT), aspartate aminotransferase (AST), tumor necrosis factor α (TNF-α), interleukin 6 (IL-6), copper and zinc superoxide dismutase (Cu–Zn-SOD), GPx, triglycerides (TG) and low-density lipoprotein (LDL) induced by ethanol. Capsicum can also increase the activity of high density lipoprotein (HDL), manganese superoxide dismutase (Mn-SOD) and CAT, suggesting that the antihypertensive mechanism of pepper may be related to the reduction of the levels of pro-inflammatory cytokines and the inhibition of oxidative stress [75].

Capsaicin (Table 1) is the active ingredient of capsicum. It is commonly used in the clinical treatment of hypertension [76], diabetes [77], fatty liver [78], Alzheimer's disease [79], peripheral neuropathic pain [80], and other diseases. Multiple studies have confirmed that capsaicin has a certain antihypertensive effect. In a RCT involving 606 hypertensive patients, capsaicin significantly reduced salt preference and daily salt intake, while decreasing SBP and DBP by 8 mmHg and 5 mmHg, respectively. The mechanism of action is related to regulating the neural processing of salt in the insula and orbital frontal cortex of the human brain [81]. Capsaicin can also lower blood pressure in 2-kidney and 1-clip (2K1C) renal vascular hypertension model rats, and its antihypertensive mechanism may be related to the enhancement of protein kinase B (Akt) and eNOS phosphorylation [82]. TRPV1 channel is a ligand-gated cation channel of the Trp subfamily, and capsaicin is the most promising agonist of TRPV1 [83]. Studies have shown that capsaicin can activate TRPV1, thereby improving the function of vascular smooth muscle cell (VSMC) and reduce hypertension [84].

Numerous studies have confirmed that the antihypertensive mechanism of capsaicin is related to the inhibition of oxidative stress. Using guinea pigs fed high fat diet as experimental model, capsaicin could reduce the levels of TC, TG, LDL-C, and increase level of high density lipoprotein cholesterol (HDL-C). Capsaicin could also reduce the levels of eNOS, MDA, ET-1 and average gray value (AGV), and increase SOD and NO levels, suggesting that the antihypertensive mechanism of capsaicin is related to inhibiting oxidative stress, improving endothelial dysfunction, and reducing risk factors for hypertension [40]. In male SD rats, capsaicin can reduce left ventricular end-diastolic pressure (LVEDP), decrease myocardial infarction area, increase SOD level, and decrease MDA content. This study suggested that the antihypertensive mechanism of capsaicin may be related to the inhibition of oxidative stress and the improvement of TOD of heart caused by hypertension [41]. Capsaicin can alleviate the blood pressure elevation caused by NO synthase inhibition in SHR [42]. A study based on erythrocytes of male Wistar rats in vitro showed that capsaicin could increase GSH levels and reduce MDA and ROS levels [39].

#### Berberine

Rhizoma coptidis, also known as "wei lian", "chuan lian" and "chicken foot lian", is a perennial herb of coptidine in ranunculaceae. TCM believes that rhizoma coptidis is cold in property, very bitter in taste, and belongs to heart, stomach, liver, large intestine meridians, with the effects of clearing heat and dampness, purging fire, and detoxifying toxicosis. Rhizoma coptidis has the pharmacological effects of hypoglycemic [85, 86], anti-inflammatory [87], antifungal [88], anti-cancer [89], etc. Multiple studies have confirmed that rhizoma coptidis has a certain antihypertensive effect. Rhizoma coptidis can improve abnormal metabolism of tryptophan, pyrimidine, arachidonic acid and bile acid in SHR, inhibit proprotein convertase subtilisin/kexin 9 (PCSK9) activation, regulate electrical signals and ionic channels, promote energy metabolis signaling pathway, and interact with non-coding RNAs via targeting multiple signaling pathways such as AMPK, mechanistic target of rapamycin (mTOR), etc. This indicates that the antihypertensive mechanism is related to improving inflammatory status, regulating lipid metabolism, and combating oxidative stress [90].

Berberine (Table 1) is a kind of quaternary ammonium alkaloid isolated from rhizoma coptidis, and is the main effective component of rhizoma coptidis[91]. Clinically, it is often used to treat hypertension [92], diabetes [93], coronary heart disease (CHD) [94], hypercholesterolemia [26, 95–97], cancer [98], irritable bowel syndrome [99] and other diseases. Berberine has a certain antihypertensive effect. A systematic review of 614 hypertensive patients, including 5 RCTs and 2 non-RCTs, showed that berberine can lower blood pressure without causing cardiovascular or long-term adverse events [92]. Berberine can not only reduce SBP by 20 mmHg and DBP by 10 mmHg, but also reduce blood glucose in diabetic rats model. Its antihypertensive mechanism is related to the activation of BKCA channel in VSMC. This study suggested that berberine can treat both hypertension and hyperglycemia simultaneously [43]. Berberine can also regulate endothelial microparticles (EMPs) and endothelial progenitor cells (EPCs) in SHR model, indicating that the antihypertensive effect may be related to improving endothelial function and reducing arterial wall stiffness [45]. Berberine can inhibit endothelial cell proliferation and inflammatory response by inhibiting the phosphorylation of PI3K/Akt, ERK1/2 and p38MAPKs signal transduction pathways, suggesting that berberine has antihypertensive and vascular protective effects [100]. In vitro experimental studies have shown that inhibiting ACE activity and promoting direct release of NO/cGMP in vascular tissue were the antihypertensive mechanism of berberine [101]. In addition, berberine can directly induce vasodilation of rabbit by inhibiting transient receptor potential vanilloid 4 (TRPV4), thereby reducing blood pressure and increasing elasticity of vascular wall [102].

The antihypertensive mechanism of berberine is related to the inhibition of oxidative stress. Berberine not only enhances the bioavailability of NO, inhibits the expression of NADPH oxidase and ROS production, but also forms stable free radicals with ROS-derived NADPH oxidase, which could reduce blood pressure [103]. Using rats with 2K1C renal vascular hypertension as experimental model, long-term administration of berberine could reduce FRA-like activity and norepinephrine (NE) levels in the paraventricular nucleus of hypothalamus (PVN), decrease the levels of NOX2, NOX4, ERK1/2 and iNOS in PVN and increase the Cu/Zn-SOD level in PVN. It suggested that berberine can alleviate hypertension and sympathetic excitability of model rats through ROS/ERK1/2/iNOS pathway [44]. Berberine can also reduce carotid cyclooxygenase-2 (COX-2) levels by activating AMPK, reducing endothelial dependent contraction, inhibiting endoplasmic reticulum stress, and clearing ROS in SHR. The hypotensive mechanism of berberine is related to the inhibition of oxidative stress and the reduction of vascular damage caused by hypertension [45]. Another in vitro study showed that berberine can activate the AMPK signaling cascade, promote phosphorylation of endothelial nitric oxide synthase (eNOS), increase NO level, reduce production of ROS induced by high glucose, inhibit activation of NK-κB and expression of adhesion molecules, and induce endothelium-dependent vasodilation. The antihypertensive mechanism of berberine is related to its inhibition of oxidative stress and protection of vascular endothelium [104].

### Puerarin

Pueraria is the dried root of the leguminous plant Kudzu. According to TCM theory, pueraria is cool in property, sweet and spicy in taste, and belongs to the lung and stomach meridians. It has the effects of relieving muscle, reducing fever, relieving rash, promoting fluid production, quenching thirst, promoting yang production and stopping diarrhea. Pueraria has the pharmacological effects such as antihypertension [105], antioxidant [106], anti-inflammatory [107], correcting insulin resistance (IR) [108], improving brain circulation [109], improving cardiac hypertrophy [110], etc. Multiple studies have shown that puerariae has a certain antihypertensive effect. Using SHR as the experimental model, 180 mg/kg flavonoids extracted from pueraria could reduce SBP by 19 mmHg and DBP by 9 mmHg after 29 days of intervention. The mechanism is associated with inhibition of ACE activity, inhibition of RAAS [111], regulation of pathways such as Nrf2 and NOX, as well as reduced activation of hypoxia-inducible factor-1α(HIF-1α), NLR pyrin domain-containing protein 3 (NLRP3), and nuclear factor-kappa (NF-κB) [112]. Taking L-NAME induced hypertensive rats as the experimental model, intervention of 60 g/kg of pueraria for 4 weeks can reduce SBP by 36 mmHg, and increase the levels of NO, eNOS and SOD in plasma. The study suggested that the antihypertensive mechanism of pueraria is related to its antioxidant effect [113].

Puerarin (Table 1) is the main active component of pueraria [114]. Modern pharmacological studies have shown that puerarin can be widely used to treat hypertension, diabetes [115], liver fibrosis [116], ischemic stroke [117], and other diseases. Puerarin has a certain antihypertensive effect. Puerarin could decrease SBP and DBP by 16.86 mmHg and 9.62 mmHg, respectively in SHR. The antihypertensive mechanism was related to the increase of NO, cGMP levels and phosphorylated eNOS protein levels, and the decrease of AT1 level [118]. Puerarin (30 mg/kg/d) intervention for 4 weeks can reduce SBP by 20 mmHg and MAP by 10 mmHg in hypersaline-induced hypertensive mice, suggesting that the hypotensive mechanism may be related to the activation of TRPV4 channel and the induction of endothelium-dependent vasodilation of mesenteric arteries in mice [49]. In addition, puerarin can reduce abnormal sympathetic activation caused by myocardial ischemia by regulating P2X3 receptors in the upper cervical ganglion of rats, thereby lowering blood pressure and heart rate [119].

Many studies have confirmed that antioxidant stress is a possible mechanism by which puerarin lowers blood pressure. In a RCT involving 160 patients with obesity hypertension, the combination of puerarin and conventional Western antihypertensive medicine could reduce MAP by 25 mmHg. Puerarin can also reduce the levels of MDA, ET-1, VWF, and thromboxin (TXB2), increase the levels of SOD, NO, and 6-keto-prostaglandin F1A (6-Keto-PGF1A), and enhance endothelial-dependent and non-endothelial-dependent vasodilation [120]. In AngII-induced hypertensive rats, puerarin can reduce SBP by 18 mmHg, and its antihypertensive mechanism may be related to thedecrease in NADPH oxidase activity, upregulation of eNOS level, and inhibition of the expression of inflammatory cytokine VCAM1. In addition, puerarin can down-regulate the levels of TGF-β1 and fibronectin in aorta to prevent and reverse cardiovascular remodeling [47]. Puerarin can not only reduce SBP and DBP by 19 mmHg and 26 mmHg respectively, but also reduce the serum MDA level and increase the levels of NO and eNOS in SHR. Its hypotensive and antioxidant stress mechanisms are related to the regulation of PI3K/Akt pathway [46]. Another study showed that puerarin can reduce SBP by 10 mmHg, and its mechanism is related to inhibiting the protein expression of NADPH oxidase gp91phox and p22phox subunit, decreasing of the p-eNOS level induced by AngII, and promoting the production of NO in blood vessels. In addition, puerarin can also improve cardiac hypertrophy and vascular hypertrophy induced by AngII, and reverse TOD induced by hypertension [121]. In vitro cell experiments, puerarin could promote the proliferation of HUVEC, inhibit ROS-induced apoptosis of endothelial cells and mitochondrial damage, indicating that the antihypertensive mechanism of puerarin may be related to the inhibition of ROS-mediated vascular endothelial dysfunction and oxidative stress [48].

#### Tanshinone IIA

Salvia miltiorrhiza is the dry root and rhizome of Salvia miltiorrhiza, which belongs to the genus Salvia in the labiaceae. According to TCM theory, Salvia miltiorrhiza is slightly cold in property, bitter in taste, and has the functions of activating blood circulation, removing blood stasis, regulating menstruations, relieving pain, clearing the heart and eliminating vexation. Salvia miltiorrhiza has the effects of anti-thrombosis [122], anti-oxidant [123], protecting vascular endothelium [124], protecting heart target organ [95], inhibiting vascular remodeling [125], etc. Multiple studies have confirmed that Salvia miltiorrhiza has a certain antihypertensive effect. An experimental study on AngII-induced hypertensive mice showed that the hypotensive mechanism of Salvia miltiorrhiza was related to the inhibition of ACE and AngII activities [126]. In the 2K1C model rats, intervention of Salvia miltiorrhiza extract (150 μg/ mL) for 3 weeks could reduce SBP by about 15 mmHg, inhibit ACE activity in a dose-dependent manner, and reduce plasma ALD level, indicating that its antihypertensive mechanism is related to the inhibition of ACE and RAAS [127]. Danshensu (10 mg/kg/d) intervention for 6 weeks can not only reduce SBP and DBP by 29 mmHg and 16 mmHg respectively, but also reduce the incidence of ventricular tachycardia and ventricular fibrillation and increase the activities of NO and iNOS [128].

Tanshinone IIA (Table 1) is the main active component of Salvia miltiorrhiza [129]. Clinically, it is often used in the treatment of hypertension [130], cardiac fibrosis [131], chronic kidney disease [132], chronic hepatitis [133] and other diseases. Numerous studies have confirmed that tanshinone IIA has a certain antihypertensive effect. A systematic review of 1696 hypertensive nephropathy patients in 16 RCTs showed that tanshinone IIA not only reduced SBP and DBP by 6.54 mmHg and 4.14 mmHg, respectively, but also lowered 24-h urinary protein and serum creatinine levels. It is suggested that tanshinone IIA can enhance the antihypertensive and renal protective efficacy of ARB drugs [134]. Tanshinone IIA can also inhibit the proliferation of rat basilar artery smooth muscle cells by inactivating 3' -phosphoric estrogen-dependent kinase, thereby exerting a role in lowering blood pressure and improving cerebral vascular remodeling caused by hypertension [135]. Tanshinone IIA can regulate the transcription level of MMPs/TIMPs balance, thereby modulating collagen metabolism and alleviating cardiac fibrosis in 2K2C rats model [136, 137]. In addition, tanshinone IIA can inhibit the Cys-C/Wnt signaling pathway and MEK/ERK pathway [138, 139], thereby improving cardiac hypertrophy.

Recent studies have shown that the antihypertensive mechanism of tanshinone IIA is related to the inhibition of oxidative stress. A RCT involving 80 patients with essential hypertension showed that combined with conventional western medicine treatment, tanshinone IIA could not only reduce SBP and DBP by 21 mmHg and 21 mmHg, respectively, but also reduce ET-1 and increase NO levels [140]. This indicated that hypotensive mechanism is related to inhibition of oxidative stress and vascular endothelial functionprotection [141]. Another RCT of 85 patients with hypertensive intracerebral hemorrhage showed that tanshinone IIA as adjuant therapy could not only improve the clinical symptoms, quality of life and self-care scores (ADL scores), but also reduce serum IL-6 and matrix metalloproteinase-9 (MMP-9) levels and neurological deficit scores (NIHSS scores) [142]. Using chronic intermittent hypoxia (CIH) -induced left ventricular dysfunction to simulate the CIH in patients with OSA syndrome, tanshinone IIA can inhibit the increase of blood pressure induced by CIH, while increasing eNOS levels and decreasing ET-1 expression. Studies have also shown that tanshinone IIA can not only reduce blood pressure, but also inhibit oxidative stress and reduce oxidative stress damage of the heart [143]. Using hypertensive rats induced by high salt feeding as the experimental model, intervention with tanshinone IIA for 16 weeks can not only reduce MAP by 22 mmHg, but also reduce the expression of Gp91phox subunit on NADPH oxidase in PVN, decrease renal sympathetic nerve discharge, reduce the level of interleukin (IL) 1β in the PVN and increase the level of IL-10 in the PVN. The antihypertensive mechanism of tanshinone IIA is related to the inhibition of oxidative stress and sympathetic excitation in PVN, and the restoration of the balance between inflammatory cytokines and anti-inflammatory cytokines [50].

#### Ligustrazine

Chuanxiong is the dried rhizome of Ligusticum chuanxiong in Umbelliform family. According to TCM theory, chuanxiong is warm in property and pungent in taste. It belongs to the liver, gallbladder and pericardium meridians, and has the effect of promoting blood circulation and qi, dispelling wind and relieving pain. Modern pharmacological studies have shown that chuanxiong is often used in the treatment of hypertension [107], vascular dementia [144], acute kidney injury [145], and osteoarthritis [146]. Many studies have confirmed that chuanxiong has a certain antihypertensive effect. The hypotensive mechanism of chuanxiong is related to the regulation of HIF-1 signaling pathway, calcium signaling pathway, VEGF signaling pathway, PI3K-Akt signaling pathway, cGMP-PKG signaling pathway, estrogen signaling pathway and others [147]. In vivo and in vitro studies have shown that the hypotensive mechanism of chuanxiong is related to its vasodilation effect, which may involve the NO/ SGC /cGMP pathway and the ATP-sensitive K + channel [107].

Ligustrazine (Table 1) is the main active ingredient of chuanxiong [148]. Pharmacological studies showed that ligustrazine has the effects of antihypertensive [149], anti-oxidant [150, 151], reducing myocardial injury [152], diastolic the pulmonary artery [153], antiplatelet aggregation [154], etc. Previous studies have shown that ligustrazine has a certain antihypertensive effect. A RCT of 110 patients with gestational hypertension showed that combined with conventional western antihypertensive drugs, administration of ligustrazine for 2 weeks could reduce SBP and DBP by 34 mmHg and 28 mmHg, respectively, and improve the hemodynamics of the patients' placental bed artery [155]. Another RCT study of 158 patients with early type 2 diabetes mellitus and hypertension with normal renal function has shown that combined with conventional treatment of western medicine, administration of ligustrazine could not only reduce SBP and DBP by 29 mmHg and 20 mmHg, respectively, but also reduce fasting blood glucose, 2-h postprandial blood glucose, BUN, Cr and GFR levels. It is suggested that ligustrazine can reduce hypertension risk factors and renal damage while lowering blood pressure [156]. An study based on porcine coronary endothelial cells (PCECs) in vitro showed that ligustrazine can regulate the expression of soluble epoxyhydrolase (sEH) by inhibiting mitochondrial stress, and protect vascular endothelial function against AngII [157]. An in vitro experimental study based on isolated rat thoracic aortic ring specimens has shown that ligustrazine has a dilatation effect on the vascular ring preconstricted by noradrenaline (NE) in a concentration-dependent manner. The vasodilation effect of ligustrazine may be one of its hypotensive mechanisms [158].

Numerous studies have confirmed that the antihypertensive mechanism of ligustrazine is related to the inhibition of oxidative stress. Experimental studies based on the two-kidney and two-clamp (2K2C) stroke-prone nephrovascular hypertension rat model (RRSP) showed that ligustrazine can down-regulate the levels of ET-1 and AngII, up-regulate the levels of NO, and improve the basilar artery remodeling in RRSP rats. The antihypertensive mechanism of ligustrazine is related to the inhibition of oxidative stress and the improvement of TOD of brain caused by hypertension [51]. Ligustrazine intervention can also reduce the SBP by 39 mmHg in SHR and increase the plasma levels of NO and GSH [52]. In vitro studies revealed that ligustrazine can inhibit hydrogen peroxide (H2O2)-induced ROS production, and has certain scavenging ability on NO, hydroxyl radical (.OH), (H2O2), DPPH radical (.DPPH) and lipid peroxidation, which could inhibit oxidative stress and protect vascular endothelium [53].

### Single Chinese herb

#### Uncaria

Uncaria (Table 2) is a dry hookrattan branch with hookrattan for Rubiaceae plants, hookrattan with large leaves, hookrattan with hair, hookrattan with Chinese hookrattan or hookrattan without stalk. In TCM theory, uncaria is cool in property and sweet in taste, which belongs to the liver and pericardium meridians. It has the effect of calming liver wind, relieving convulsion, and clearing heat. Modern pharmacological studies showed that uncaria has the effects of antihypertensive [159], diastolic blood vessels, protecting the cranial nerves, anti-inflammatory [160, 161], etc. Clinically, it is often used to treat hypertension [162], Alzheimer's disease [163], Parkinson's disease [164], anxiety disorders [165], depression [166] and other diseases.

**Table 2:** Protective effects of single Chinese herb in the regulation of oxidative stress for hypertension

Single Chinese herb	Effects	Active ingredients	Animal/cells	Targets	Refs.
*Gouteng* (Uncaria)	Calming liver wind, relieving convulsion, and clearing heat	Rhynchophylline, isorhynchophylline, total alkaloids of uncaria and isodehydrorhynchophylline	SHR (introvenous injection, 20 mg/kg, 4 h)	RAAS↓, SNS↓, AngII↓, IL-6↓, IL-1β↓, TNF-α↓, SOD↑, NO↑, NOS↑	[167]
*Duzhong* (Eucommia ulmoides)	Nourishing liver and kidney, strengthening muscles and bones, and promoting fetal safety	lignans, pinoresinol diglucoside, etc	SHR (oral administration, 200, 600 and 1200 mg/kg/d, 22 days) and HUVEC (incubate with ox-lDl, 100,250,500 and 1000 μg/, 24 h)	eNOS↓, NO↑	[168, 169]
*Huangqi* (Astragalus)	Tonifying qi and raising yang, strengthening the surface, preventing perspiration, tonifying the spleen, benefiting the lung, reducing swelling and reducing water, supporting toxin and raising muscle	Astragaloside I, II, III, IV (Astragaloside A), capsule Astragaloside I, II, etc	Salt-sensitive hypertensive rat (intragastric administration, 1.32 and 1.68 g/kg^−1^/d, 4 weeks)	MDA↓, SOD↑, GSH-Px↑	[170]
*Shihu* (Dendrobium)	Nourishing yin, clearing heat, benefiting stomach and promoting the secretion of saliva or body fluid	Polysaccharides, flavonoids, alkaloids, etc	SHR (oral administration, 4.50 g/kg/d, 8 weeks)	TC↓, TG↓, LDL-C↓, HDL-C↑, Glu↓, INS↓, CRP↓, IL-6↓, AngII↓, ET-1↓, TXB2↓, PGI2↑, PI3K/Akt/eNOS↓, GSH-Px↑, CAT↑, SOD↑, NO↑, eNOS↑	[171]

Multiple studies have confirmed that uncaria has a certain antihypertensive effect. Uncaria could decrease SBP by 30 mmHg, lower facial temperature, and increase pain threshold in model rats. The mechanism might be related to the up-regulation of the contents of dihydrotestosterone, 16a hydroxy-androsterone, deoxycorticosterone and 2-methoxyestrone, while downregulating the L-tryptophane level [166]. In pregnant rats, uncaria can not only reduce SBP by 33 mmHg, but also reduce the mean 24 h proteinuria, and decrease the levels of IL-6, IL-1β, TNF-α and interferon-γ in serum and placenta. The antihypertensive mechanism of uncaria is related to anti-inflammatory effect [172]. Rhynchophylline, isorhynchophylline, total alkaloids of uncaria and isodehydrorhynchophylline are the main antihypertensive components of uncaria [173]. Isorhynchophylline can reduce SBP by 25 mmHg in SHR, and its hypotensive mechanism is related improving hypothalamic neurotransmitter imbalance and inhibiting excessive activation of RAAS and SNS [174]. Using N' -nitrol-L-arginine induced hypertensive rats as experimental model, total alkaloids of uncaria can reduce SBP by 30 mmHg, DBP by 29 mmHg and MAP by 30 mmHg after 5 weeks of intervention. It can also protect vascular endothelial cells and reduce the loss of endothelial cells. The mechanism is related to reducing overall low-grade inflammation [175]. Total alkaloid of Uncaria can improve the sensitivity of SHR artery baroreceptor reflex, and improve the target organ scores of heart, brain and kidney for hypertension [176]. In addition, at 0.5 h, 1 h, 2 h, 3 h and 4 h after the intervention of rhynchophylline, the SBP decreased rates of model rats were 7.2%, 12%, 19.2%, 21.6%, 21.2%, and the DBP decreased rates were 5.7%, 13.3%, 20.9%, 24.1%, 24.3%, respectively. Rhynchophylline can also dilate the coronary arteries and basilar arteries of model rats [177].

Multiple studies have confirmed that the hypotensive mechanism of uncaria is related to the inhibition of oxidative stress. Combined with western antihypertensive therapy, rhynchophylline can reduce SBP by 17 mmHg in SHR, decrease the plasma AngII level and increase the serum NO and NOS content, suggesting that the mechanism of uncaria in treating hypertension is related to protecting vascular endothelium and inhibiting oxidative stress [167]. In addition, uncaria can not only reduce SBP, but also down-regulate the levels of MDA, and TNF-α inflammatory factor, and increase the content of SOD. Uncaria can also improve renal interstitial fibrosis and collagen deposition, protect the kidney and inhibit oxidative stress in rats [178].

#### Eucommia ulmoides

Eucommia ulmoides (Table 2) is the bark of the Eucommia ulmoides plant. Eucommia ulmoides is warm in property, sweet in taste, slightly pungent, and belongs to liver and kidney meridians, with the effects of nourishing liver and kidney, strengthening muscles and bones, and promoting fetal safety [179]. Eucommia ulmoides has the effects of antihypertensive, hypoglycemic, anti-cancer, anti-aging, diastolic blood vessels [180, 181], etc. Clinically, it is often used to treat hypertension, rheumatoid arthritis, diabetes, and hyperlipidemia [182, 183].

Numerous studies have confirmed that Eucommia ulmoides has a certain antihypertensive effect. A RCT of 96 hypertensive patients showed that compared with western antihypertensive drugs alone, the combination treatment of Eucommia ulmoides leaf decoction slices for 30 days could lower SBP by 17 mmHg and DBP by 12 mmHg, and lower the incidence of adverse reactions such as dizziness, headache and allergic reaction. The study suggested that Eucommia ulmoides could enhance the antihypertensive effect of conventional western antihypertensive drugs [184]. Intervention with Eucommia ulmoides male flower intervention for 7 weeks can reduce SBP in a dose-dependent manner, and its hypotensive mechanism is related to the activation of ACE2-Ang-(1-7)-Mas signaling pathway [185]. Eucommia ulmoides can prevent and reverse renal damage caused by hypertension by improving glomerular sclerosis and alleviating structural changes of anterior glomerular vessels [186]. Eucommia Bark is the main medicinal part of Eucommia ulmoides. Eucommiae Folium treats hypertension through a multi-target and multi-pathway mechanism including epithelial sodium channel, heat shock protein 70, rho-associated protein kinase 1, catalase, and superoxide dismutase. The relevant signal transduction pathways included the ras homolog family member A (RhoA)/Rho-associated protein kinase (ROCK) and nicotinamide adenine dinucleotide phosphate (NADPH) oxidase/eNOS/NO/Ca2 + pathways [187]. Eucommia ulmoides exerts a hypotensive effect by improving the morphology of colon tissue in SHR, improving intestinal microbiota imbalance, and altering fecal metabolism patterns [188]. Eucommia ulmoides extract at a dose of 600 mg/kg/d or 1200 mg/kg/d for 22 days can reduce the blood pressure at a rate of about 10 mmHg/h starting from the 8th day in SHR [168]. Intervention of Eupermoides ulmoides extract for 17 weeks can not only reduce the SBP of model rats by about 15 mmHg, but also lower the body weight and blood adiponectin levels, and improve the thickening of aortic medium in high-fat diet fed rats. Another study suggested that Eupermoides ulmoides can not only lower blood pressure, but also improve the risk factors of hypertension and protect target organs [189]. The hypotensive mechanism of Eucommia ulmoides is related to the inhibition of aldose reductase activity, which could prevent and reverse hypertensive heart remodeling [190].

The antihypertensive mechanism of Eucommia ulmoides is related to the inhibition of oxidative stress. Ulcommia ulmoides can reduce MAP by 35 mmHg and decrease the ratio of superior mesenteric artery wall thickness (WT) to vessel diameter (LD), which may be related to the inhibition of aldose reductase activity and the reversing of vascular remodeling [191]. Eucommia ulmoides could inhibit ox-LDL-induced eNOS uncoupling and up-regulate NO levels in HUVEC, indicating that its antihypertensive mechanism may be related to inhibiting oxidative stress and improving endothelial dysfunction [169].

#### Astragalus

Astragalus (Table 2) is the root of the legumes Astragalus mongolicus and Astragalus membranaceus. According to TCM theory, Astragalus is mild in property, sweet in taste, and belongs to the lungs, spleen, liver and kidney meridians. It has the functions of tonifying qi and raising yang, strengthening the surface, preventing perspiration, tonifying the spleen, benefiting the lung, reducing swelling and reducing water, supporting toxin and raising muscle. Modern pharmacological studies have confirmed that Astragalus has the effects of antihypertensive, anti-inflammatory [192], strengthening the immune [193], anti-oxidant [194], antiviral [195], reversing cardiac hypertrophy [196], inhibition of oxidative stress, fibrosis, endoplasmic reticulum stress, apoptosis, and ferroptosis, as well as the regulation of autophagy[197], etc. Clinically, it is often used to treat hypertension [198], pulmonary hypertension [199], diabetes [200], malignant tumor [201]and other diseases.

Multiple studies have confirmed that astragalus has a certain antihypertensive effect. A systematic review of 429 hypertensive patients with renal damage in 5 RCTs showed that astragalus injection could reduce β2 microglobulin, microalbuminuria, BUN, and Ccr, suggesting that astragalus injection could protect renal damage [202]. A RCT involving 154 postmenopausal women with hypertension and metabolic syndrome revealed that astragalus can improve the left ventricular early diastolic mitral velocity (E'), the ratio of early diastolic peak mitral velocity to late diastolic peak mitral velocity (E/A), the ratio of E' to late diastolic peak mitral velocity (E'/A') and the ratio of early diastolic peak mitral velocity (E) to E' (E/E') and alleviate left ventricular diastolic dysfunction with a dose-dependent manner. Astragalus can improve diastolic function and protect target organs in hypertensive patients [203]. Another RCT of 156 patients with senile hypertension showed that intervention of astragalus for 6 weeks can increase the elasticity of large and small arteries, reduce the vascular overload index, reduce the level of vascular injury indexes such as ET-1 and vWF, and reduce the left ventricular remodeling indexes such as left ventricular mass index, left ventricular end-diastolic volume, and left ventricular end-systolic volume. In brief, astragalus has a certain protective effect on heart and blood vessels [204].

Numerous studies have shown that the antihypertensive mechanism of astragalus is related to the inhibition of oxidative stress. A RCT involving 180 patients with renal hypertension has shown that astragalus intervention for 20 days could not only lower SBP and DBP by 62 mmHg and 28 mmHg, respectively, but also lower serum MDA level, and increase SOD, NO and NOS levels in patients with renal hypertension [205]. Another RCT of 82 patients with essential hypertension showed that intervention of astragalus injection for 14 days could increase the serum expression levels of TAC, CAT and GSH-Px, reduce the serum expression levels of AOPP, decrease the serum expression levels of ET-1, sVCAM-1 and vWF, decrease the levels of VIO and baPWV, and increase the level of FMD. This indicates that the hypotensive mechanism of astragalus is related to enhancing antioxidant capacity and improving vascular function [206]. Astragalus ultrafiltration substance can lower average blood pressure, improve the thickening of thoracic aorta, reduce the content of MDA, and increase the activities of SOD and GSH-Px in salt-sensitive hypertensive rats [170]. The mechanism of lowering blood pressure and reversing vascular remodeling may be related to the inhibition of oxidative stress. Astragalus can reduce MDA level and inhibit enzymatic and non-enzymatic lipid peroxidation [207]. In vitro experiments showed that astragaloside IV could exert vasodilation effect through the endothelium-dependent NO-cGMP pathway. In the presence of perivascular fat, astragaloside IV could inhibit the vasoconstriction induced by deoxyadrenaline and angiotensin II by blocking extracellular Ca^2+^ influx. The study suggested that regulation of vasomotor function might be one of the hypotensive mechanisms of astragalus [192].

#### Dendrobium

Dendrobium (Table 2) is the stem of the orchid plants Dendrobium annulus, Dendrobium verbena, Dendrobium officinale, Dendrobium yellow-grass, or Dendrobium officinale. In TCM theory, dendrobium is slightly cold in property, sweet in taste, and belongs to stomach and kidney meridians, with the effects of nourishing yin, clearing heat, benefiting stomach and promoting the secretion of saliva or body fluid [208]. Modern pharmacological research has shown that dendrobium officinale has effects such as lowering blood pressure, blood sugar [209], lipid [210], and uric acid [211], antioxidant [212, 213], and immune regulation [214]. Clinically, it is often used in the treatment of hypertension, diabetes [215], malignant tumor [216], diabetic cardiomyopathy [217], fatty liver disease [218], postmenopausal osteoporosis [219] and other diseases.

Previous studies have confirmed that dendrobium has a certain antihypertensive effect. A RCT involving 120 hypertensive patients showed that compared with conventional western antihypertensive drugs alone, dendrobium officinalis could significantly improve the antihypertensive efficiency by 20%, and the scores of symptoms such as vertigo, headache, chest tightness, tinnitus and so on were significantly increased [220]. In metabolic hypertensive rats, intervention with dendrobium officinale ultramicropowder for 15 weeks can not only reduce the SBP, DBP and MAP of the model rats by 20 mmHg, 10 mmHg and 20 mmHg, respectively, but also significantly reduce TC, TG, LDL-C, Glu and insulin (INS) levels. Other researches also showed that dendrobium can improve glucolipid metabolism and inhibit inflammatory response associated with hypertension by increasing the level of HDL-C and reducing the levels of endotoxin, CRP and IL-6 [221]. Intervention with 4.50 g/kg Dendrobium officinale for 8 weeks resulted in a decrease of 28 mmHg in SBP and 14 mmHg in DBP, respectively. And no significant increase in blood pressure was observed after 1 week of withdrawal in SHR. In addition, dendrobium officinale could also reduce plasma AngII content in a dose-dependent manner [171]. Intervention with 1.0 g/kg Dendrobium for 28 days can not only reduce SBP by 16 mmHg, but also reduce cardiac weight index and left ventricular hypertrophy index (LVHI). The cardioprotective mechanism of dendrobium is related to improving the arrangement of myocardial myofibrils, reducing mitochondrial damage and autophagosome formation, indicating that dendrobium can lower blood pressure and delay the progression of hypertensive left ventricular hypertrophy [222]. In vivo and in vitro experiments have shown that dendrobium can significantly inhibit p-ERK protein expression, reduce left ventricular systolic blood pressure (LVSP), cardiac natriuretic hormone and brain natriuretic hormone levels, prevent and reverse cardiac hypertrophy [223].

The antihypertensive mechanism of dendrobium may be related to the inhibition of oxidative stress. Dendrobium can not only reduce the SBP, DBP and MAP by 14 mmHg, 9 mmHg and 11 mmHg, respectively, but also inhibit the thickening of thoracic aorta and the loss of endothelial cells, reduce the plasma contents of ET-1 and TXB2, and increase the levels of PGI2, NO and eNOS in hypertensive rats induced by high glucose and high fat combined with alcohol. The hypotensive mechanism of dendrobium is related to inhibiting oxidative stress, protecting vascular endothelium, and improving vascular dilation [224]. Intervention with Dendrobium for 4 weeks can reduce the SBP, DBP and MAP by 6 mmHg, 4 mmHg and 5 mmHg respectively in "eating disorder" hypertensive rats induced by high sugar, high fat diet and alcohol. Its antihypertensive mechanism is related to the activation of PI3K/Akt/eNOS signaling pathway, the increase of NO level, and the decrease of intercellular adhesion molecule-1 and ET-1 levels, which could improve the ability of anti-oxidative stress [225]. In the rats with metabolic hypertension induced by compound diet, intervention with 2 g/kg ethanol extract of dendrobium officinale for 6 weeks could lower SBP by 8 mmHg, reduce MDA level and increase GSH-Px level in rats with metabolic hypertension induced by compound diet [226]. In addition, dendrobium can also increase the levels of SOD and CAT in SHR, which could enhance the anti-oxidative stress ability [227].

### TCM classic formula

#### Tianma Gouteng decoction

Tianma Gouteng decoction (Table 3) originates from *Hu Guangci*'s "New Meaning of Treating Miscellaneous Diseases and Syndrome in Internal Medicine of Traditional Chinese Medicine" (《杂病证治新义》) during the Republic of China. It is composed of gastrodia elata, uncaria, concha haliotidis, eucommia ulmoides, achyranthes root, mistletoe, gardenia, scutellaria baicalensis, motherwort, poria cocos, and tuber fleeceflower stem. Tianma Gouteng decoction has the effects of calming the liver and suppressing wind, clearing heat, promoting blood circulation, and tonifying the liver and kidneys, which is often used in the treatment of liver yang hyperactivity syndrome such as headache, dizziness, tinnitus, insomnia, dreaminess, bitter mouth, blushing, tongue redness, yellow coating, and string pulse. In modern times, it is often used to treat diseases such as hypertension [228], stroke [229], migraine [230], Alzheimer's disease [231], and Parkinson's disease [232].

**Table 3:** Protective effects of TCM classic formula and traditional Chinese patent medicine in the regulation of oxidative stress for hypertension

Formulas	Main components	Effects	Animal/cells	Targets	Refs.
*Tianma Gouteng* decoction	Gastrodia elata, uncaria, concha haliotidis, eucommia ulmoides, achyranthes root, mistletoe, gardenia, scutellaria baicalensis, motherwort, poria cocos, and tuber fleeceflower stem	Calming the liver and suppressing wind, clearing heat, promoting blood circulation, and tonifying the liver and kidneys	SHR (oral administration, 40 g/kg/d, 4 weeks)	OPG↑, TRAIL↑, RAAS↓, Cu–Zn SOD↑	[233, 234]
*Banxia Baizhu Tianma* decoction	Pinellia ternata, atractylodes macrocephala, gastrodia elata, poria cocos, radix liquiritiae, dried tangerine peel, jujube, and ginger	Resolving phlegm, dispelling wind, strengthening spleen and clearing dampness	Obesity-related hypertension mice (oral administration, 8.64 g/kg/d, 20 weeks), SHR (oral administration, 8.64 g/kg/d, 13 weeks)	TC↓, TG↓, Glu↓, IL-1↓, IL-6↓, TNF-α↓, MAPK↓, iNOS↓, NO↑	[235], [236]
*Huanglian Jiedu* decoction	Coptis coptis, Scutellaria scutellaria, Phellodendron amurense and Fructus Gardeniae	Clearing away heat and toxic material	SHR (oral administration, 2.5 and 5.0 g/kg/d, 28 days)	Slc4a1↑, Ppp1r3d↑, Rrm2↑, Pim1↑, Kcnh1↓, Nr4a3↓, ET-1↓, MDA↓, SOD↑, GSH-Px↑, NOS↑	[237]
*Songling Xuemaikang* capsule	Pine leaves, kudzu root, pearl powder and other medicines	Calming the live, suppressing yang, calming the heart and nerves, promoting blood circulation and removing blood stasis	SHR (oral administration, 708.75 mg/kg/d, 2 weeks)	CaMKIIδ↓, ERK1/2/GATA4↓, RAAS↓, ALD↑, PBEF↓, IL-6↓, JAK/STAT↓, ERK/MEK↓, NO↑, SOD↑, GSHPx↑	[238, 239]
*Tongxinluo* capsule	Leech, ginseng, scorpion, radix paeoniae rubra, soil turtle, borneol, cicada slough and other medicines	Replenishing qi, activating blood, removing blood stasis and relieving pain	Carotid artery ligation mice (oral administration, 0.75 g/kg^−1^/d), human cardiac microvascular endothelial cells (200, 400 and 600 μg/ml^−1^, 24 h), SHR (oral administration, 1.0 g/kg/d, 8 weeks)	Akt1↑, AngII↓, ET-1↓, VEGF↓, vWF↓, TBF-α↓, IL-6↓, SOD↑, NO↑, GSH-Px↑, CAT↑, MDA↓	[240]; [241, 242]
*Yangxue Qingnao* granule	Chinese angelica, Chuanxiong, Radix Paeoniae Rubra, Rehmannia glutinosa, Uncaria, Caulis spatholobi, Prunella vulgaris, Cassia Seed, Mother of Pearl, Corydalis, Asarum and other medicines	Nourishing blood, calming the liver, promoting blood circulation and clearing collaterals	SHR (oral administration, 0.5 g/kg/d, 4 weeks), RHR (oral administration, 5 g/kg/d, 4 weeks)	Src/MLCK/MLC↓, ET↓, NO↑	[243–245]

Numerous clinical and experimental studies have shown that Tianma Gouteng decoction has a certain antihypertensive effect. A systematic review of 1808 hypertensive patients including 22 RCTs showed that, on the basis of conventional western antihypertensive medicine, Tianma Gouteng decoction can not only improve the clinical symptoms such as headache, dizziness, insomnia, palpitation, tinnitus, irritability, but also lower SBP and DBP by 6.71 mmHg and 4.6 mmHg respectively [246]. A RCT of 251 patients with masked hypertension showed that Tianma Gouteng granules could lower daytime SBP by 5.44 mmHg and daytime DBP by 3.39 mmHg for 4 weeks, and no serious adverse effect was observed [247]. Another RCT of 80 hypertensive patients with liver yang hyperactivity syndrome showed that on the basis of conventional western antihypertensive drugs, Tianma Gouteng decoction could reduce SBP and DBP by 37 mmHg and 21 mmHg, respectively, decrease the level of ET-1 in vascular endothelium and increase the content of NO, indicating a protective effect on vascular endothelial injury [248]. Intervention of Tianma Gouteng decoction with 40 g/kg/d for 4 weeks could lower SBP, DBP and MAP by about 20 mmHg, 10 mmHg and 10 mmHg respectively in SHR. In addition, it It has a cardiovascular protective effect by up-regulate the protein expression of osteoprotegerin (OPG) and TNF-related apoptosis-inducing ligands (TRAIL), and activate p-Akt, inhibit the caspase cascade reaction, and thereby inhibit the apoptosis of cardiomyocytes [234]. Another experimental study suggested that MAP could be decreased by 24 mmHg after 0.5 mL/100 g body weight intervention of Tianma Gouteng decoction for 2 weeks in 5-week-old SHR, and the hypotensive mechanism may be related to the regulation of sympathetic activity and vasomotor function [233]. *Tianma Gouteng* decoction can also lower blood pressure through improving the levels of AngII, renin and aldosterone (ALD), which could inhibit RAAS activity [233]. In addition, Tianma Gouteng decoction contains active components that enhance memory ability, which can prolong the stepping latency and protect brain function in ICR rats [249]. Intervention of Tianma Gouteng granules with 10 mL/kg for 14 days can regulate glucose metabolism in amygdala, left thalamus and other brain functional areas, and enhance the activity of corresponding brain functional areas and alleviating cognitive dysfunction caused by hypertension [250].

The antihypertensive mechanism of Tianma Gouteng decoction may be related to the inhibition of oxidative stress. A meta-analysis of 1510 hypertensive patients including 14 RCTs showed that Tianma Gouteng decoction could lower plasma endothelin level, improve NO level and SOD activity, while lowering blood pressure, suggesting the antihypertensive mechanism may be related to inhibition of oxidative stress and protection of vascular endothelium [251]. A recent study further found that the inhibition of oxidative stress and inflammation may be related to the upregulation of transcription factor EB (TFEB) expression by Tianma Gouteng decoction [252]. It has been found that the active ingredient baicalein in Tianma Gouteng decoction can effectively increase the activities of SOD and GSH-Px in rat tissue [253]. This study suggests that the antioxidant activity of baicalein may be at least partially due to its inhibition of ROS formation derived from ET-1/ETA. Tianma Gouteng decoction can also regulate PC12 cells differentiated from hypoxic glucose neurons and the antioxidant system and anti-apoptotic genes in rats with middle cerebral artery occlusion [254], suggesting that the antihypertensive mechanism may be related to inhibiting oxidative stress and protecting the brain nerves. Additionally, Tianma Gouteng decoction could significantly improve the distaxation degree of superior mesenteric artery, and up-regulate the levels of Cu–Zn SOD, 4-α-methotrexate dehydratase 1 and arginine dimethylaminohydrolase 2 in SHR. This study suggested that the antihypertensive mechanism may be related to the inhibition of oxidative stress and the improvement of vasomotor function [255].

#### Banxia Baizhu Tianma decoction

Banxia Baizhu Tianma decoction (Table 3) originates from Medical Heart Enlightenment (《医学心悟》) written by *Cheng Guo-peng* in *Qing* Dynast, which could resolving phlegm, dispelling wind, strengthening spleen and clearing dampness. It is composed of pinellia ternata, atractylodes macrocephala, gastrodia elata, poria cocos, radix liquiritiae, dried tangerine peel, jujube, and ginger. It is commonly used for wind-phlegm disturbance syndrome. Clinically, it is often used for the treatment of hypertension [256], hyperlipidemia [257], neurovertigo [258].), vertebrobasilar artery insufficiency vertigo [259] and other diseases.

Numerous clinical and experimental studies have shown that Banxia Baizhu Tianma decoction has a certain antihypertensive effect. A systematic review of 1424 patients with essential hypertension including 16 RCTs showed that Banxia Baizhu Tianma decoction can enhance the antihypertensive effect of conventional western antihypertensive drugs [260]. A RCT of 56 patients with H-type hypertension revealed that, on the basis of conventional western medicine treatment, Banxia Baizhu Tianma decoction could lower SBP and DBP by 27 mmHg and 15 mmHg, respectively, and lower Hcy level [261]. Experimental studies have also shown that Banxia Baizhu Tianma decoction can improve vascular remodeling, ventricular hypertrophy, and cardiovascular risk factors. Intervention of Banxia Baizhu Tianma decoction for 8 weeks could not only reduce SBP and DBP by 12.1 mmHg and 10.5 mmHg, respectively, but also lower the body weight and TC in obesity-related hypertension mice induced by high fat diet. In addition, in vitro experimental studies have shown that Banxia Baizhu Tianma decoction can reverse ox-LDL-induced vascular endothelial injury, suggesting the hypotensive mechanism may be related to the protective effect of vascular endothelial [235]. Intervention of Banxia Baizhu Tianma decoction of 8.64 g/kg for 13 weeks could not only lower SBP by 25 mmHg, but also improve the left ventricular mass index in SHR, suggesting the protective effect of heart damage caused by hypertension. Its hypotensive and cardioprotective effects may be related to the decreasing of IL-1, IL-6, TNF-α and iNOS levels, and the inhibition of inflammatory response [236]. Intervention of Banxia Baizhu Tianma decoction of 13.8 g/(kg·d) for 12 weeks could not only reduce SBP and DBP by 24 mmHg and 22 mmHg respectively, but also reduce the body weight and the levels of serum glucose, TC and TG in rats [262].

In addition, it has been identified that the antihypertensive mechanism of Banxia Baizhu Tianma decoction may be related to the inhibition of oxidative stress. Intervention of Banxia Baizhu Tianma decoction of 13.8 g/kg/d for 12 weeks could not onlylower SBP and DBP by 45 mmHg and 25 mmHg respectively, but also reduce the end diastolic septal thickness, the left ventricular posterior wall thickness and the left ventricular mass index, increase the end diastolic diameter of the left ventricle, and the level of NO in SHR. The mechanism of action may be related to reducing the activity of mitogen-activated protein kinase (MAPK) signaling pathway and enhancing the ability of antioxidant stress [263]. In vivo and in vitro experimental studies showed that Banxia Baizhu Tianma decoction could not only lower blood pressure in SHR, but also play a vasodilator role mediated by NO/SGC/cGMP cascade, prostacyclin (PGI2), mycarinic pathway and calcium channel. The antihypertensive mechanism maybe related to the inhibition of oxidative stress and the improvement of vascular activity [264]. The RCT of 100 hypertensive patients showed that, on the basis of conventional antihypertensive medicine, Banxia Baizhu Tianma decoction can improve the level of SOD, reduce the MDA, 8-hydroxy acid deoxyguanosine, and UA [265].

#### Huanglian Jiedu decoction

Huanglian Jiedu decoction (Table 3) derives from Essential Secrets from Outside the Metropolis by Wang Tao in Tang Dynasty, which is composed of Coptis coptis, Scutellaria scutellaria, Phellodendron amurense and Fructus Gardeniae [266]. It has the effect of clearing away heat and toxic material, and is used in the treatment of intense heat in the tri-jiao, with symptoms such as severe fever, irritability, dry mouth, dry throat, wrong speech, insomnia, vomiting blood, blemishes, hot spots, diarrhea, damp-heat jaundice, carbuncle, furuncle, red and yellow urine, red tongue, yellow coating, and strong pulse. Commonly, Huanglian Jiedu decoction is used in modern clinical treatment of hypertension, hepatocellular carcinoma [267], diabetes [268, 269], ischemic stroke [266, 270], sepsis [271], ulcerative colitis [272] and other diseases.

Huanglian Jiedu decoction has a certain antihypertensive effect. A RCT involving 90 patients with refractory hypertension showed that compared with conventional antihypertensive drug, Huanglian Jiedu decoction can effectively reduce SBP and DBP by 53 mmHg and 33 mmHg, respectively. The mechanism may be related to the reduction of plasma endothelin (ET) level and upregulation of calcitonin gene-related peptide (Calcitonin gene related peptide, CGRP) level [96]. Huanglian Jiedu decoction can regulate glucose metabolism, lower blood pressure, and transform risk factors for hypertension. A RCT involving 98 hypertensive patients has shown that compared with conventional antihypertensive drug, Huanglian Jiedu decoction can not only lower SBP and DBP by 8.5 mmHg and 7.1 mmHg respectively, but also reduce fasting blood glucose and fasting insulin levels [273]. Animal experiment has shown that intervention of Huanglian Jiedu decoction for 7 weeks can reduce SBP by about 25 mmHg in SHR. Its mechanism is related to the up-regulation of gene expression such as Slc4a1, Ppp1r3d, Rrm2, Pim1, and down-regulation of gene expression such as Kcnh1, Nr4a3, etc. [237]. In addition, Huanglian Jiedu decoction can alleviate the pathological myocardial hypertrophy and remodeling caused by transverse aortic constriction. The potential therapeutic mechanism is to regulate lipid metabolism such as cholesterol, ceramides, fatty acids and phospholipids. The antihypertensive mechanism of Huanglian Jiedu decoction may be related to improving lipid metabolism [274].

The antihypertensive mechanism of Huanglian Jiedu decoction is related to the inhibition of oxidative stress. A RCT involving 90 patients with hypertension and hyperlipidemia showed that Huanglian Jiedu decoction can not only lower blood pressure and lipids, but also reduce serum MDA and ET-1 levels, increase SOD and GSH-Px activity and overall antioxidant capacity of the body [275]. Huanglian Jiedu decoction can lower ET-1 and von Willebrand factor (von Willebrand Factor, vWF) levels, and increase NOS and calmodulin levels, the antihypertensive mechanism of which is related to inhibiting oxidative stress and improving vascular endothelial function [274].

### Traditional Chinese patent medicine

#### Songling Xuemaikang capsule

Songling Xuemaikang capsule (Table 3) is composed of pine leaves, kudzu root, pearl powder and other medicines. It has the effects of calming the live, suppressing yang, calming the heart and nerves, promoting blood circulation and removing blood stasis. It is used for headaches, dizziness, irritability, palpitations and insomnia caused by hyperactivity of liver yang, hypertension [276] and primary hyperlipidemia with the syndromes above.

Songling Lingxuemaikang capsule has a certain antihypertensive effect, and can enhance the effect of antihypertensive drugs. A systematic review of 1778 patients with essential hypertension including 17 RCTs has shown that on the basis of conventional antihypertensive drugs, Songling Xuemaikang capsule can further lower SBP and DBP by 6.17 and 7.24 mmHg respectively [277]. A RCT involving 60 hypertensive patients with liver-yang hyperactivity syndrome has shown that Songling Xuemaikang capsule can not only improve insomnia, dizziness, headache, irritability, hot flashes, night sweats and other hypertension-related symptoms, but also reduce SBP and DBP by 9 mmHg and 7 mmHg respectively [278]. A RCT involving 96 hypertensive patients showed that compared with conventional antihypertensive drugs alone, Songling Xuemaikang capsule can improve the circadian rhythm and blood pressure variability [188]. Preventing and reversing cardiac hypertrophy and delaying the process of hypertension related cardiac damage may be one of potential antihypertensive mechanisms of Songling Xuemaikang capsule. Using rats with isoproterenol-induced cardiac hypertrophy as experimental model, it can improve the diastolic thickness of the posterior wall of the left ventricle and inhibit ISO-induced cardiac hypertrophy in a dose-dependent manner. Its mechanism of action is related to the inhibition of CaMKIIδ and ERK1/2/GATA4 signaling pathway [188, 279]. Studies have shown that the antihypertensive mechanism of Songling Xuemaikang capsule is related to regulating the activity of the RAAS system and inhibiting inflammation. It can lower SBP and DBP by 15 mmHg and 10 mmHg, respectively, and its mechanism may be related to the reduction of plasma AngII levels in SHR [188]. In addition, intervention with Songling Xuemaikang capsule of 708.75 mg/kg for 2 weeks can not only lower SBP and DBP by 17 mmHg and 35 mmHg respectively, but also improve the plasma ALD level, down-regulate the expression of PBEF, IL-6 and other inflammatory response-related genes, and inhibit JAK/STAT and ERK/MEK mediated cascade reactions in SHR [239].

The hypotensive mechanism of Songling Xuemaikang capsule may be related to the inhibition of oxidative stress. A RCT involving 112 patients with H-type hypertension as well as carotid artery stenosis showed that, in addition to conventional treatment, intervention of Songling Xuemaikang capsule for 3 months can not only lower SBP and DBP by 38 mmHg and 22 mmHg, respectively, but also improve Hcy, TC, LDL-C and ET levels, and increase the levels of NO, SOD and GSHPx. The hypotensive mechanism of Songling Xuemaikang capsule is related to inhibiting oxidative stress, dilating blood vessels, and regulating lipid metabolism [280]. A RCT involving 94 hypertensive patients with liver-yang hyperactivity syndrome showed that on the basis of conventional antihypertensive drugs, Songling Xuemaikang capsule for 4 weeks can lower 24 h SBP and DBP by 29 and 20 mmHg, reduce 24 h average SBP and DBP variability by 7 and 5 mmHg, decrease MDA level, and increase NOS and SOD levels [281]. Intervention of Songling Xuemaikang capsule of 1 g/kg for 9 weeks can reduce the MDA concentration and increase the NO concentration in SHR, suggesting that Songling Xuemaikang capsule can reduce the oxidative stress damage. And its mechanism of action may be related to the down-regulation of CAV1 gene expression and up-regulation of IGF1R gene expression [238].

#### Tongxinluo capsule

Tongxinluo capsule (Table 3) is mainly composed of leech, ginseng, scorpion, radix paeoniae rubra, soil turtle, borneol, cicada slough and other medicines. It has the functions of replenishing qi, activating blood, removing blood stasis and relieving pain [282]. It is used for the treatment of coronary heart disease, angina pectoris, with the syndrome of deficiency of heart-qi, blood stasis and collateral obstruction. Clinical application indications include chest tightness, tingling, colic, palpitations, spontaneous sweating, shortness of breath, fatigue, purplish tongue, and thready and uneven pulse.

Tongxinluo capsule has certain antihypertensive effects. A systematic review of 1958 hypertensive patients including 25 RCTs has shown that, on the basis of conventional antihypertensive drugs, the antihypertensive effect of Tongxinluo capsule was significantly better than antihypertensive drugs alone [283]. A RCT involving 100 patients with hypertension and coronary heart disease showed that on the basis of conventional antihypertensive drugs, Tongxinluo capsule can reduce SBP and DBP by 31 and 16 mmHg, respectively, improve cardiac function classification and 6-min walking distance [284]. Researches have suggested that Tongxinluo capsule can reduce brain damage caused by hypertension. Tongxinluo capsule administered 24 h after cerebral infarction can promote ipsilateral thalamic neurogenesis and angiogenesis in model rats, and improve neuronal function in middle cerebral artery occlusion model of renovascular hypertensive rats [285]. The vasoprotective effect may be an important mechanism of Tongxinluo capsule for hypertension. Using carotid artery ligation mice as an experimental model, Tongxinluo capsule can increase the expression and phosphorylation of Akt1 protein in bone marrow macrophages stimulated by TNF-α, thereby inhibiting the expression of miR-155 and blocking the feedback loop between miR-155 and TNF-α, thereby inhibiting vascular inflammation and preventing vascular remodeling [241]. In vitro experimental studies also showed that the antihypertensive mechanism of Tongxinluo capsule may be related to the inhibition AngII activity and improvement of vascular endothelial function. Tongxinluo capsule can up-regulate tight junction proteins occludin, claudin, VE-cadherin and β-catenin induced by AngII in human cardiac microvascular endothelial cells. The mechanism involves inducing the expression of Krüppel-like factors 5 in microvascular endothelial cells [240].

The antihypertensive mechanism of Tongxinluo capsule may be related to the inhibition of oxidative stress. A RCT involving 174 patients with hypertension and type 2 diabetes has shown that, in addition to significantly reducing SBP and DBP, Tongxinluo capsule can also reduce fasting blood glucose and 2 h postprandial blood glucose levels, increase SOD, NO, GSH-Px and CAT levels, and decrease MDA and ET-1 levels [244, 245]. A RCT involving 82 hypertensive patients showed that on the basis of conventional antihypertensive drugs, Tongxinluo capsule can significantly reduce VEGF, vWF, ET-1 levels and increase NO levels while reducing blood pressure [286]. Experimental studies have shown that Tongxinluo capsule can protect SHR from kidney damage caused by hypertension by inhibiting oxidative stress, anti-fibrosis and anti-inflammatory activities. The intervention of Tongxinluo capsule for 12 weeks can increase creatinine clearance of SHR, reduce urine albumin, reduce glomerular sclerosis, renal podocyte damage and tubular interstitial fibrosis, reduce MDA levels and increase manganese Manganese superoxide dismutase (Mn-SOD) and CAT levels. It can also inhibit the expression of TBF-α and IL-6. The potential mechanism may be related to the activation of Forkbox O1 signaling function [242]. Using homocysteine thiolactone 50 mg/kg/d in rats fed for 8 weeks as an experimental model, intervention of Tongxinluo capsule of 1.0 g/kg/d can significantly inhibit oxidative stress and improve the endothelial function of rats. Its mechanism of action involves PPARγ-dependent oxidative stress inhibition. In addition, in vitro experimental studies have also shown that Tongxinluo capsule can reverse the decrease in SOD activity and the increase in MDA content in rat aortic tissue induced by homocysteine thiolactone, suggesting that the antihypertensive mechanism may be related to the inhibition of oxidative stress and protection of vascular endothelium [287].

#### Yangxue Qingnao granule

Yangxue Qingnao granule (Table 3) is mainly composed of Chinese angelica, Chuanxiong, Radix Paeoniae Rubra, Rehmannia glutinosa, Uncaria, Caulis spatholobi, Prunella vulgaris, Cassia Seed, Mother of Pearl, Corydalis, Asarum and other medicines. It has the effects of nourishing blood, calming the liver, promoting blood circulation and clearing collaterals. It is often used for headache, dizziness, upset, irritability, insomnia and dreaminess caused by blood deficiency and liver yang hyperactivity syndrome. Currently, it is often used to treat hypertension, Alzheimer's disease [288], chronic cerebral insufficiency [289] and other diseases.

Yangxue Qingnao granule has a certain antihypertensive effect [290]. Numerous systematic evaluations have shown that Yangxue Qingnao granule, as a complementary therapy, have a synergistic antihypertensive effect. A systematic review of 1985 patients with essential hypertension including 12 RCTs has shown that compared with antihypertensive drugs alone, Yangxue Qingnao granule combined with antihypertensive western medicine has a more obvious effect on lowering SBP and DBP, suggesting that Yangxue Qingnao granule can enhance the clinical efficacy of antihypertensive drugs [291]. A systematic review and sequential analysis of trials that included 34 RCTs and a total of 4532 patients with essential hypertension has shown that, on the basis of conventional antihypertensive drugs, combined use of Yangxue Qingnao granule can not only lower SBP and DBP by 10.56 mmHg, and 8 0.21 mmHg respectively, but also improve the clinical symptoms including chest tightness, palpitations, memory loss with no serious adverse effects [243]. A RCT involving 96 hypertensive patients has shown that on the basis of conventional antihypertensive western medicines, combined with Yangxue Qingnao granules for 4 weeks can not only reduce SBP by 15 mmHg, DBP by 7 mmHg, 24 h SSD by 4 mmHg, 24 h DSD by 3 mmHg, but also improve dizziness, headache, shortness of breath, fatigue, insomnia and other clinical symptoms [292]. Yangxue Qingnao granule could lower blood pressure, which could prevent and reverse hypertensive brain damage. Intervention of Yangxue Qingnao pill by 0.5 g/kg/d for 4 weeks can not only lower the MBP by about 30 mmHg, SBP by about 35 mmHg, and DBP by about 20 mmHg in SHR, but also reduce the cerebral blood flow in the hippocampus and cortex, reduce the permeability of the brain blood vessels, reduce brain tissue edema, and inhibit neuronal apoptosis in hippocampus and cortex. And its mechanism may be related to the inhibition of Src/MLCK/MLC signaling pathway [244, 245].

Previous studies have shown that the antihypertensive mechanism of Yangxue Qingnao granule is related to the inhibition of oxidative stress. Using Renovascular hypertension rat (RHR) as experimental model, intervention of Yangxue Qingnao granule of 5 g/kg for 4 weeks can reduce the SBP of RNR by about 15 mmHg, while reducing plasma ET content, increasing the content of NO and calcitonin genes related peptides [243].

## 4. Discussion

The importance of oxidative stress in the pathogenesis of hypertension is gradually receiving attention. Firstly, the pathogenesis of hypertension is related to various factors, such as endothelial damage, abnormal activation of the RAAS system, and abnormal activation of the SNS system (Fig. 1). Oxidative stress can act on these mechanisms, promoting the occurrence and development of hypertension and its related TOD. Oxidative stress induced endothelial damage is a key mechanism in the occurrence and development of hypertension. Secondly, the occurrence and development of hypertension involve abnormalities in various signaling pathways, including the PI3K/Akt/eNOS pathway, Notch3 signaling pathway, and NF-κB pathway, MAPK pathway, aortic transforming growth factor (TGF)-β1 pathway, vascular endothelial growth factor (VEGF) pathway, AMPK/mTOR pathway, etc. Oxidative stress can affect the aforementioned signaling pathways, leading to hypertension. In addition, hyperlipidemia, diabetes, homocysteine and other risk factors will also aggravate oxidative stress, further promoting the occurrence and development of hypertension and TOD.

However, previous researches on oxidative stress and hypertension have mostly been limited to experimental studies, epidemiological investigations, and retrospective studies. Currently, there is a lack of evidence-based evidence to support the use of antioxidant stress therapy for hypertension. Some design shortcomings were existed in the previous RCT studies, including small sample size, inadequate research methods, and lack of long-term follow-up. Therefore, there is a lack of high-quality clinical evidence. In addition, although numerous studies have shown that some drugs such as vitamin C and vitamin E have certain antioxidant effects, there is currently a lack of evidence to suggest that these drugs treat hypertension by inhibiting oxidative stress. Therefore, current single target therapy has limitations and the clinical translation of antioxidant drugs is insufficient [293, 294].

The clinical application of TCM has a history of more than 2000 years and is widely used in the treatment of cardiovascular diseases [295]. Oxidative stress has become an important target and mechanism for the treatment of hypertension with TCM.

Firstly, TCM has the advantages of multi-target, multi-component, and overall comprehensive regulation. In the treatment of hypertension, it not only has antihypertensive effect, but also improves hypertension related clinical symptoms and quality of life, reverses risk factors, and protects target organs such as the heart, brain, and kidneys from damage. Its mechanism of action is related to comprehensive regulatory effects such as vasodilation, improvement of endothelial function, anti-inflammatory, regulation of RAAS, inhibition of sympathetic nervous system activity, and antioxidant activity [105, 296]. Recently, with the development of interdisciplinary fields, it has been identified that novel technologies such as nanoparticles and exosomes can be combined with TCM to treat hypertension. One important reason for poor blood pressure control may be the low bioavailability of drugs. The recent solution to improve dosage and bioavailability issues is to incorporate drugs into nanoparticle carriers [297]. Although no antihypertensive nanoformulations have received clinical approval so far, they have shown some potential. Nanomaterials can reduce the particle size of insoluble flavonoids, prolong the adhesion and retention time of drugs in the gastrointestinal tract, significantly improve solubility, enhance bioavailability, and solve the delivery difficulties of flavonoids, which underscored the potential of nanomaterials in exploring the medicinal value of Chinese herbal medicine resources [112]. Isoliensinine, a kind of bibenzyl isoquinoline alkaloid which isolated from Lotus Plumule (Nelumbo nucifera Gaertn), exhibits antihypertensive and vascular smooth muscle proliferation-inhibiting effects. Due to poor water solubility and low bioavailability, its clinical application is limited. Compared with free isoliensinine, both in vitro and in vivo experiments have demonstrated that isoliensinine loaded by PEG-PLGA polymer nanoparticles can effectively enhance its antihypertensive effect [298]. Another study identified that Taraxacum officinale-derived exosome-like nanovesicles were effective in the treatment of intermittent hypoxia-induced hypertensive disease, whereas butyrate played a major role in mediating the effects of exosome-like nanovesicles in anti-intermittent hypoxia-induced hypertensive disease [15].

Secondly, a large number of TCM classical herbal formulas and monomers have certain antioxidant effects, which have been confirmed by clinical and experimental studies [5, 299]. Ginsenoside Rb1, allicin, capsaicin, berberine, puerarin, tanshinone IIA and other effective ingredients, single Chinese herbal medicine such as Dendrobium, Uncaria, Eucommia, Astragalus, Tianma Gouteng decoction, Banxia Baizhu Tianma decoction, Huanglian Jiedu decoction and other classic famous prescriptions, as well as traditional Chinese patent medicines such as Songling Xuemaikang capsule, Tongxinluo capsule, Yangxue Qingnao granule, can not only reduce the SBP, DBP or MAP of hypertensive patients or hypertensive model rats, but also reduce the levels of MDA, NADPH oxidase, ET-1, and increase the levels of SOD, CAT, GSH Px, eNOS and NO.

Thirdly, the biological basis of TCM syndromes of hypertension has been a research hotspot in recent years. Studies have shown that, as the most common TCM syndrome of hypertension, the liver yang hyperactivity syndrome is closely related to oxidative stress. A study based on 448 patients showed that the levels of SOD, CAT, and GSH-Px in the liver yang hyperactivity group were significantly reduced, while the level of MDA was significantly increased. This suggested that in hypertensive patients with liver yang hyperactivity syndrome, oxidative stress function is reduced, which is involved in the formation process of TCM syndrome [300]. In another experimental study, the model of liver yang hyperactivity syndrome in SHR was successfully developed. Liver yang hyperactivity syndrome is not only characterized by markedly increased SBP, hyper-RASS system, increased urine N-acetyl-β-Dglucosidase and microalbuminuria, increased AT1R/AT2R ratio of skeletal muscle, IR, accompanied by renal and skeletal muscle damage, but also by significant upregulation of ROS/Akt signaling factor expression. Intervention with Chinese herbs with calming the liver and suppressing the hyperactive yang can significantly downregulate the expression of ROS/Akt signal factor [301].

In summary, oxidative stress is one of the important mechanisms underlying the occurrence and development of hypertension, and antioxidant stress is a new approach and target for treating hypertension. Unlike the single target therapy strategy for antioxidant stress in modern medicine, TCM has gradually gained clinical attention due to its multi-target regulatory advantages in anti hypertension and antioxidant stress damage [302]. This may have some similarities with the "cocktail" therapy of modern medicine. In future researches, it is necessary to pay more attention to the methodological quality of clinical researches in order to obtain high-quality clinical evidence of TCM for treating hypertension through antioxidant damage, and further explore its mechanism of action, providing reference for the discovery of new targets and innovative drug research in the treatment of hypertension with TCM.

## Data Availability

Data availability is not applicable to this article as no new data were created or analyzed in this study.

## Abbreviations

ACE: Angiotensin converting enzyme
ACE2: Angiotensin converting enzyme 2
ADL scores: Life and self-care scores
AG: American ginseng
AGV: Average gray value
ALD: Aldosterone
ALT: Alanine aminotransferase
Ang I: Angiotensin I
Ang II: Angiotensin II
AST: Aspartate aminotransferase
AT1R: Angiotensin II type 1 receptor
BPV: Blood pressure variability
BUN: Blood urea nitrogen
CAT: Catalase
CCR: Creatinine clearance
CGRP: Calcitonin gene related peptide
CHD: Coronary heart disease
CHM: Chinese herbal medicine
CIH: Chronic intermittent hypoxia
CKD: Chronic kidney disease
COX-2: Cyclooxygenase-2
CPSSA: S-allylmercaptocaptopril
Cu–Zn-SOD: Copper and zinc superoxide dismutase
DBP: Diastolic blood pressure
EDHF: Endothelium-derived hyperpolarizing factor
EMPs: Endothelial microparticles
eNOS: Endothelial nitric oxide synthase
EPCs: Endothelial progenitor cells
ET: Endothelin
ET-1: Endothelin1
ETAR: ETA-receptor
GSH-Px: Glutathione peroxidase
Hcy: Homocysteine
HDL: High density lipoprotein
HDL-C: High density lipoprotein cholesterol
HIF-1α: Hypoxia-inducible factor-1α
HUVEC: Human umbilical vein endothelial cells
H_2_S: Hydrogen sulfide
IL: Interleukin
IL-6: Interleukin 6
INS: Insulin
IR: Insulin resistance
KRG: Korean red ginseng
LD: Vessel diameter
LDL: Low-density lipoprotein
LDL-C: Low-density lipoprotein cholesterol
Lp-PLA2: Lipoprotein-associated phospholipase A2
LVEDP: Left ventricular end-diastolic pressure
LVHI: Left ventricular hypertrophy index
LVSP: Left ventricular systolic blood pressure
lysoPCs: Lysophosphatidylcholines
MAPK: Mitogen-activated protein kinase
MDA: Malondialdehyde
MMP-9: Matrix metalloproteinase-9
Mn-SOD: Manganese superoxide dismutase
NADPH: Nicotinamide adenine dinucleotide phosphate
NE: Norepinephrine
NIHSS: Scoresneurological deficit scores
NF-κB: Nuclear factor-kappaB
NLRP3: NLR pyrin domain-containing protein 3
NO: Nitricoxide
NO-SGC-cGMP: Nitric oxy-soluble guanidate-cyclase-cyclic guanosine phosphate
OPG: Osteoprotegerin
PCECs: Porcine coronary endothelial cells
PEG–PLGA: Polyethylene glycol–poly lactic acid-co-glycolic acid
PGI2: Prostacyclin
PGI2-ac-cAMP: Prostacyclin-adenylate cyclase-cyclic adenosine phosphate
PVN: Paraventricular nucleus of hypothalamus
RAAS: Renin–angiotensin–aldosterone system
RHR: Renovascular hypertension rat
ROS: Reactive oxygen species
SBP: Systolic hypertension pressure
SCR: Serum creatinine
sEH: Soluble epoxyhydrolas
SHR: Spontaneously hypertensive rat
SNS: Sympathetic nervous system
SOD: Superoxide dismutase
TC: Total cholesterol
TCM: Traditional Chinese medicine
TG: Triglycerides
TGF: Transforming growth factor
TNF-α: Tumor necrosis factor α
TOD: Target organ damage
TRAIL: TNF-related apoptosis-inducing ligands
TFEB: Transcription factor EB
TRPV4: Transient receptor potential vanilloid 4
TXB2: Thromboxin
VEGF: Vascular endothelial growth factor
VSMC: Vascular smooth muscle cell
vWF: Von Willebrand Factor
WT: Wall thickness
XOD: Xanthineoxidase
2K1C: 2-Kidney and 1-clip
2K2C: 2 Kidneys and 2 clamps rats
6-Keto-PGF1A: 6-Keto-prostaglandin F1A
